# Same lesion, different outcomes: hemodynamic correlates of ischemic stroke in unilateral high-grade internal carotid artery stenosis or occlusion – a retrospective transcranial Doppler study

**DOI:** 10.3389/fneur.2026.1892791

**Published:** 2026-09-01

**Authors:** Yanqiu Jia, Tiantian Huo, Mingyue Fan, Jiayu Zhang, Zhenjie Teng, Weihong Chen, Wei Jin, Yanhong Dong, Peiyuan Lv

**Affiliations:** 1 Department of Neurology, Hebei Medical University, Shijiazhuang, Hebei, China; 2 Department of Neurology, Hebei General Hospital, Shijiazhuang, Hebei, China; 3 Hebei Provincial Key Laboratory of Cerebral Networks and Cognitive Disorders, Shijiazhuang, Hebei, China; 4 Department of Neurological Rehabilitation, Hebei General Hospital, Shijiazhuang, Hebei, China

**Keywords:** cerebral hemodynamics, internal carotid artery, occlusion, stenosis, transcranial Doppler ultrasound

## Abstract

**Background:**

High-grade stenosis or occlusion of the internal carotid artery (ICA) is a leading cause of ischemic stroke (IS). However, patients with severe ICA lesions often experience varying outcomes. This study investigated the relationship between cerebral hemodynamics, as detected by transcranial Doppler ultrasound (TCD), and the occurrence of IS in patients with high-grade stenosis or occlusion of unilateral ICA.

**Methods:**

A total of 272 patients diagnosed with high-grade stenosis or occlusion of unilateral ICA were retrospectively enrolled in the study. The baseline data, laboratory examination results, TCD, duplex scan examination of neck vessels and imaging information were collected. The correlation between TCD hemodynamic parameters and IS was assessed by dividing the included patients into a stroke group and a non-stroke group according to the presence or absence of an IS at this admission. Binary logistic regression and trend tests were used to analyze the data.

**Results:**

Hemodynamic parameters measured by TCD, including ipsilateral middle cerebral artery (MCA) flow velocity, P2 segment of the posterior cerebral artery (PCA-P2) flow velocity, and reversal of ophthalmic artery flow (ROAF), were identified as factors associated with IS. In particular, lower MCA flow velocity and higher PCA-P2 flow velocity were associated with the presence of IS.

**Conclusion:**

In patients with high-grade stenosis or occlusion of unilateral ICA, hemodynamics measured by TCD is associated with the presence of IS. However, prospective studies are warranted before these TCD parameters can be applied to guide the timing of intervention for ICA lesions.

## 1 Introduction

Unilateral high-grade stenosis or occlusion of the internal carotid artery (ICA) is one of the causes of ischemic stroke (IS). Patients with unilateral high-grade stenosis or occlusion of the ICA may experience transient ischemic attacks or even severe stroke events. Although previous studies have shown that embolic mechanisms play an important role in the development of IS after high-grade stenosis of the ICA, the efficiency of the opening of the collateral circulation, especially the primary collateral branches, is an important guarantee of blood supply to the distal part of the stenosis. Although more advanced imaging techniques are now available to determine blood flow supply (1), the role of transcranial Doppler ultrasound (TCD) in the assessment of collateral circulation should not be overlooked because of its advantages of being inexpensive, reproducible, and available at the bedside. Cerebrovascular collaterals detected by TCD are associated with the occurrence and recurrence of stroke (2–4). The aim of this study was to investigate the relationship between TCD-detected hemodynamics and acute IS after high-grade stenosis or occlusion of the unilateral ICA.

## 2 Methods

### 2.1 Subjects

Patients with high-grade stenosis or occlusion of the unilateral ICA who were hospitalized in Hebei General Hospital from April 2017 to May 2023 were retrospectively included.

All TCD, MRI and duplex examinations were performed within 2 days of admission. Most IS patients were admitted on the day of symptom onset, or within 2 days of it. Thus, the time intervals between symptom onset, imaging and TCD were generally within 4 days.

Patients were screened according to the inclusion and exclusion criteria.

#### 2.1.1 Inclusion criteria

(1) Age>18 years old
(2) Definition of high-grade stenosis or occlusion of the unilateral ICA: carotid ultrasound suggests that the degree of stenosis of the extracranial segment of the ICA is 70–99% or completely occluded (5). TCD showed decreased blood flow velocity (BFV) (defined as≥30% reduction in peak systolic velocity (PSV) compared with the contralateral middle cerebral artery (MCA)), delayed spectral systolic peak and decreased pulsatility index (PI) in the affected MCA compared to the contralateral MCA.

It should be emphasised that patients with ICA stenosis of 70–99% that had not yet affected distal MCA hemodynamics were not included. Compression of the contralateral common carotid artery (CCA) resulted in decreased BFV in the affected MCA (a CCA compression testing was not routinely performed on the ipsilateral side of the lesion for safety reasons).

#### 2.1.2 Exclusion criteria

(1) Acute ICA occlusion or embolism.
(2) Acute IS in the 6 months before the current admission.
(3) Previous carotid stenting or endarterectomy, intracranial arterial stenting or brain surgery.
(4) Severe stenosis or occlusion of the ipsilateral external carotid artery (ECA), the contralateral anterior circulation and the posterior circulation.
(5) IS occurring outside the territory supplied by the ipsilateral ICA.
(6) Cardiogenic embolism, traumatic brain injury, intracranial infection, intracranial space occupation, hydrocephalus, cerebral hemorrhage, intracranial pressure changes due to massive cerebral infarction, etc.
(7) Combined with toxic encephalopathies, metabolic encephalopathies, etc.
(8) With vasculitis, arterial entrap ent, arteriovenous malformation, cerebral amyloid angiopathy, moyamoya disease, etc.
(9) The presence of fever, anemia and thyroid dysfunction, etc.
(10) With serious medical conditions such as atrial fibrillation or serious arrhythmia, cardiopulmonary, hepatic and renal dysfunction, or poor general condition.
(11) Absence of an anterior communication artery.
(12) Poor temporal bone window transillumination.

The study was conducted in accordance with the Declaration of Helsinki. The study protocol was reviewed and approved by the Ethics Committee of the Hebei General Hospital (approval number: 2024-LW-064) and informed consent was waived. All data collected and analyzed in the study were derived from clinical records, and there was no interference with clinical treatment or impact on patients’ privacy and rights.

### 2.2 Baseline information of patients

We collected the patients’ age, sex, body mass index (BMI) (kg/m^2^), smoking, alcohol consumption, blood pressure on the day of TCD [including systolic blood pressure (SBP) and diastolic blood pressure (DBP)], medical history [coronary heart disease, type 2 diabetes mellitus, hypertension, and stroke (i.e., at least 6 months ago)], and laboratory tests, including hemoglobin, triglycerides, total cholesterol, high-density lipoprotein cholesterol (HDL-C), low-density lipoprotein cholesterol (LDL-C) and very low-density lipoprotein cholesterol (VLDL-C). The stroke and non-stroke groups were divided according to whether IS occurred during the current hospitalization. The diagnostic criteria for IS were based on the *Chinese Guidelines for the Diagnosis and Treatment of Acute Ischaemic Stroke 2023* (6).

### 2.3 Transcranial Doppler ultrasound

TCD examinations are primarily performed by a neurologist who has undergone standardized training and is proficient in standard TCD operating procedures, including intracranial artery identification and blood flow spectrum acquisition. In the absence of the primary neurologist, a backup operator with equivalent training may perform the examinations. All operators follow uniform standardized protocols for probe placement, acoustic window selection, and velocity measurements.

The TCD devices used in this study were the TCD-2000 T transcranial Doppler flow analyzer (Beijing Chioy medical technology Co., Ltd., China) and the DWL Multi-Dop X transcranial Doppler diagnostic system (DWL Elektronische Systeme GmbH, Germany). Both devices were equipped with one 2 MHz pulsed-wave (PW) probe and one 4 MHz continuous-wave (CW) probe. Uniform parameter settings (including sample volume, gain, filter, scale, and sweep speed) were applied to both devices. Prior to data collection, we regularly performed comparative tests on the same volunteers using both devices and confirmed that the flow velocity measurements showed good consistency, thereby ensuring the comparability and reliability of all data.

A complete TCD examination was performed in supine position within 48 h of hospital admission according to the *Practice Standards for Transcranial Doppler Ultrasound published in 2007* (7). The arteries of interest included the ipsilateral MCA, posterior cerebral artery P2 segment (PCA-P2) and ophthalmic artery (OA).
The MCA (blood flow towards the probe, depth 45-55 mm) and the PCA-P2 (probing angle of 10–30° posteriorly, blood flow away from the probe, accompanied by the basal vein, depth 55-75 mm) were probed with a 2-MHz hand-held probe through the temporal window. If the MCA BFV was reduced when the contralateral CCA was compressed, this indicated that the MCA was accurately probed and the anterior communicating artery was open. BFV data were recorded, including PSV, mean flow velocity (MFV), and end-diastolic velocity (EDV). The PI of the MCA was calculated using the formula PI = (PSV-EDV)/MFV.
The OA was probed vertically (40-50 mm depth) through the orbital window using a 2-MHz hand-held probe. The direction of OA blood flow was recorded: normal direction (i.e., towards the probe) or reverse ophthalmic artery blood flow (ROAF). Place a 4 MHz handheld probe at the inner canthus and slowly move it along the probe until a blood flow signal is detected (typically a branch of the OA—the supratrochlear artery (STA)). The ipsilateral branches of the ECA (superficial temporal and facial arteries) were simultaneously compressed to the bone surface with the thumb and index finger, respectively, and changes in STA blood flow were observed (8). A reduction in blood flow velocity or a reversal of flow direction in the STA can further confirm the presence of ROAF.

### 2.4 Duplex scan examination of the cervical arteries

Duplex ultrasonography of the carotid arteries was performed using EPIQ7C (Philips Inc., Amsterdam, The Netherlands). The degree of stenosis in the extracranial segment of the ICA arteries was graded according to ultrasound criteria, based on the NASCET arterial stenosis grading method (5).

### 2.5 Brain MRI

Brain MRI was performed with a standard protocol using 3.0 Tesla MRI scanners (Sigma, GE healthcare of America). This included at least MRA and diffusion tensor imaging (DWI).

### 2.6 Statistical analysis

SPSS 21.0 (IBM, Corporation, Armonk, NY) was used for data analysis. Continuous variables were expressed as mean ± SD or median (interquartile range) using a Kolmogorov-Smirnova test. Independent samples *t*-test or Mann–Whitney U test was used to compare between two groups as appropriate. Categorical variables were expressed as the number of cases (percentage) and comparisons between groups were made using the chi-squared test. Variables were selected for the multivariable model based on univariate *p* < 0.1, while clinically important variables were forced into the model regardless of their *p*-values (MFV was used for MCA and PCA-P2 BFV data). Tolerance and variance inflation factor (VIF) in the diagnosis of covariance were used to determine the multicollinearity of each influential factor. The presence of covariance was defined as tolerance < 0.1 and VIF > 10.

The medians of the tertiles of the MFV in the MCA and PCA-P2 were tested for trend as a continuous variable. *p <* 0.05 was considered statistically significant. We further performed subgroup-specific multivariable binary logistic regression analyses for patients with high-grade ICA stenosis and those with complete ICA occlusion, respectively.

## 3 Results

### 3.1 Participant characteristics

In total, 272 eligible patients were enrolled. The characteristics of all participants are shown in Table 1. The mean age of the participants was 64 (56, 70.75) years, and 87.9% were male (*n* = 239). Although all patients had high-grade stenosis or occlusion of the unilateral ICA, 161 were diagnosed with IS and included in the stroke group, and 111 were included in the non-stroke group. In the stroke group, a higher proportion of patients smoked (*p =* 0.046) and consumed alcohol (*p* = 0.001), had a history of hypertension (*p =* 0.049), and had higher DBP (*p =* 0.009). Patients in the stroke group had a significantly higher prevalence of prior stroke compared with the non-stroke group (*p* < 0.001). The stroke group had a lower HDL-C levels (*p* = 0.047).

**Table 1:** Baseline characteristics and TCD parameters of the study subjects.

Variable	Total (*n* = 272)	Stroke group (*n* = 161)	Non-stroke group (*n* = 111)	Statistical value *t, χ2, Z*	*p*-value
Age, median (IQR), years	64 (56, 70.75)	65 (56,71)	62 (55, 70)	−1.237	0.216
Sex (male), *n* (%)	239 (87.9)	144 (85.6)	95(89.4)	0.916	0.338
BMI, median (IQR), kg/m^2^	25.19 (23.67, 27.20)	25.23 (23.66, 27.13)	25.1 (23.84, 27.27)	−0.501	0.616
Smoking, *n* (%)	135(49.6)	88 (54.7)	47 (42.3)	3.987	**0.046**
Alcohol use, *n* (%)	70 (25.7)	53 (32.9)	17 (15.3)	10.653	**0.001**
Hypertension, *n* (%)	210 (77.2)	131 (81.4)	79 (71.2)	3.881	**0.049**
SBP, median (IQR), mmHg	139 (128, 154.75)	140 (130, 156)	135 (125, 148)	−1.958	**0.0502**
DBP, median (IQR), mmHg	80 (72, 89)	81 (73, 91)	78 (70, 86)	−2.612	**0.009**
History of coronary heart disease, *n* (%)	62 (22.8)	41 (25.5)	21 (18.9)	1.6	0.206
History of stroke, *n* (%)	52 (19.1)	49 (30.4)	3 (2.7)	32.678	**<0.001**
T2DM, *n* (%)	91 (33.5)	54 (33.5)	37 (33.3)	0.001	0.972
Complete occlusion of unilateral ICA, *n* (%)	133 (48.9)	84 (52.2)	49 (44.1)	1.695	0.193
Laboratory examination					
HGB, median (IQR), g/L	139.5 (132, 149)	139 (132.5, 149.5)	140 (127, 149)	−0.389	0.697
Total triglyceride, median (IQR), mmol/L	1.2 (0.91, 1.62)	1.22 (0.92, 1.7)	1.17 (0.89, 1.55)	−1.078	0.281
Total cholesterol, median (IQR), mmol/L	4.09 (3.34.4.82)	4.07 (3.44, 4.7)	4.10 (3.21, 5.04)	−0.569	0.569
HDL-C, median (IQR), mmol/L	1.01 (0.87, 1.16)	1.00 (0.86, 1.12)	1.04 (0.88, 1.23)	−1.987	**0.047**
LDL-C, median (IQR), mmol/L	2.57 (2.06, 3.2)	2.57 (2.15, 3.09)	2.58 (1.93, 3.45)	−0.328	0.743
VLDL-C, median (IQR), mmol/L	0.42 (0.29, 0.59)	0.42 (0.29, 0.59)	0.42 (0.29, 0.59)	−0.109	0.913
TCD parameters					
MCA PSV, median (IQR), cm/s	61.5 (47.25, 76.75)	56 (44, 72)	65 (52, 80)	−3.304	**0.002**
MCA MFV, median (IQR), cm/s	43 (34, 54)	40 (32, 52)	47 (38, 58)	−3.118	**0.002**
MCA EDV, median (IQR), cm/s	35 (27.25, 44)	32 (26, 43)	37 (31, 47)	−3.056	**0.002**
MCA PI, median (IQR)	0.58 (0.48, 0.7)	0.58 (0.48, 0.69)	0.6 (0.48, 0.70)	−0.533	0.594
PCA-P2 PSV, median (IQR), cm/s	105 (69, 139.75)	111 (69.5, 152)	94 (68, 123)	−2.728	**0.006**
PCA-P2 MFV, median (IQR), cm/s	66 (44.83, 88.91)	70 (48.67, 99.83)	61.33 (44, 77.67)	−2.78	**0.005**
PCA-P2 EDV, median (IQR), cm/s	46 (32.25, 65.75)	50 (35, 73)	43 (31, 55)	−2.673	**0.008**
ROAF, n (%)	223 (82)	141 (87.6)	82 (73.9)	8.354	**0.004**

IQR, interquartile range; BMI, body mass index; SBP, systolic blood pressure; DBP, diastolic blood pressure; T2DM, type 2 diabetes mellitus; ICA, internal carotid artery; HGB, hemoglobin; HDL-C, high density lipoprotein cholesterol; LDL-C, low density lipoprotein cholesterol; VLDL-C, very low density lipoprotein cholesterol; MCA, middle cerebral artery; PCA-P2, posterior cerebral artery P2 segment; PSV, peak systolic velocity; MFV, mean flow velocity; EDV, end-diastolic velocity; ROAF, reversal of ophthalmic artery flow. Bold values indicate statistical significance (*p* < 0.05).

### 3.2 Association between MCA MFV and IS

MCA MFV was lower in the stroke group (*p* = 0.002), whereas there was no statistically significant difference between the two groups with respect to PI (*p* = 0.594; Table 1). In unadjusted binary logistic regression analysis, MCA BFV was associated with the presence of IS in patients with unilateral high-grade stenosis or occlusion of the ICA (OR: 0.975, 95% CI: 0.960–0.991, *p* = 0.002). In a multifactorial binary logistic regression analysis, the association remained significant after adjustment for confounding factors (OR: 0.973, 95% CI: 0.955–0.992, *p* = 0.006; Table 2).

**Table 2:** The binary logistic regression analyzes between possible variables and stroke.

Variables	Univariate analysis		Multivariable analysis*	
	OR (95% CI)	*p*-value	OR (95% CI)	*p*-value
Age	1.015 (0.990–1.041)	0.240	1.016 (0.983–1.050)	0.356
Sex	0.701 (0.338–1.455)	0.340	0.840 (0.324–2.177)	0.719
Smoking	1.642 (1.008–2.674)	0.046	1.179 (0.588–2.367)	0.643
Alcohol use	2.714 (1.471–5.005)	0.001	3.302 (1.443–7.611)	0.005
Hypertension	1.769 (0.999–3.131)	0.050	1.364 (0.680–2.738)	0.382
SBP	1.011 (0.999–1.024)	0.075	0.997 (0.979–1.016)	0.784
DBP	1.026 (1.006–1.048)	0.013	1.030 (1.000–1.061)	0.047
History of stroke	15.750 (4.766–52.047)	<0.001	14.835 (4.049–54.359)	<0.001
T2DM	1.009 (0.605–1.685)	0.972	1.325 (0.690–2.542)	0.398
Complete occlusion of unilateral ICA	1.380 (0.849–2.244)	0.193	1.399 (0.757–2.586)	0.284
HDL-C	1.025 (0.942–1.115)	0.573	1.026 (0.937–1.122)	0.583
MCA MFV	0.975 (0.960–0.991)	0.002	0.973 (0.955–0.992)	0.006
PCA-P2 MFV	1.013 (1.005–1.022)	0.002	1.016 (1.006–1.027)	0.002
ROAF	2.493 (1.326–4.688)	0.005	3.931 (1.764–8.760)	<0.001

OR, odds ratio; CI, confidence interval; SBP, systolic blood pressure; DBP, diastolic blood pressure; T2DM, type 2 diabetes mellitus; HDL-C, high density lipoprotein cholesterol; MCA, middle cerebral artery; PCA-P2, posterior cerebral artery P2 segment; MFV, mean flow velocity; ROAF, reversal of ophthalmic artery. * Adjusted for variables in the table.

There was a significant trend between higher MCA MFV and a lower prevalence of IS in patients with unilateral high-grade stenosis or occlusion of the ICA. In the unadjusted test for trend, the highest tertile of MCA MFV (≥50.1; median 61.0) was significantly associated with a reduced presence of stroke in patients with unilateral high-grade stenosis or occlusion of the ICA (OR: 0.421, 95% CI: 0.228–0.780, *p* for trend = 0.010). After adjustment for all the confounding factors, the OR for the highest versus lowest tertile of the MCA MFV was 0.403 (95% CI: 0.197–0.828; *p* for trend = 0.033; Table 3).

**Table 3:** ORs (95%CIs) of stroke according to tertiles of MCA MFV.

Model	MCA MFV (cm/s), median (range)			*p* for trend
	Tertile1 31.0 (≤37.0)	Tertile2 43.0 (37.1–50.0)	Tertile3 61.0 (≥50.1)	
Model 1	Reference	0.460 (0.249–0.848)	0.421 (0.228–0.780)	0.010
Model 2	Reference	0.470 (0.203–0.816)	0.493 (0.250–0.972)	0.082
Model 3	Reference	0.344 (0.163–0.727)	0.403 (0.197–0.828)	0.033

Model 1: Unadjusted.

Model 2: Adjusted for age, sex, smoking, alcohol use, hypertension, DBP, history of stroke and complete occlusion of unilateral ICA.

Model 3: Adjusted for age, sex, smoking, alcohol use, hypertension, DBP, history of stroke and complete occlusion of unilateral ICA, PCA-P2 MFV and ROAF.

OR, odds ratio; CI, confidence interval; MCA, middle cerebral artery; PCA-P2, posterior cerebral artery P2 segment; MFV, mean flow velocity; ROAF, reversal of ophthalmic artery flow.

### 3.3 Association between PCA-P2 MFV and IS

PCA-P2 MFV was higher in the stroke group than in the non-stroke group (*p* = 0.005; Table 1). In unadjusted binary logistic regression analysis, increased PCA-P2 MFV was strongly associated with the presence of IS in patients with unilateral high-grade stenosis or occlusion of the ICA (OR: 1.013, 95% CI: 1.005–1.022, *p* < 0.001). In a multifactorial binary logistic regression analysis, the association remained significant after adjustment for confounding factors (OR: 1.016, 95%CI: 1.006–1.027, *p* = 0.002; Table 2).

The test for trend showed that the presence of IS increased with higher PCA-P2 MFV. After adjustment for all confounding factors, the highest tertile of the PCA-P2 MFV (≥81.1; median 103.0) versus the lowest tertile (≤53.3; median 37.3) had an OR of 3.651 (95% CI: 1.772–7.741; *p* for trend<0.001; Table 4).

**Table 4:** ORs (95%CIs) of stroke according to tertiles of PCA-P2 MFV.

Model	PCA-P2 MFV (cm/s), median (range)			*p* for trend
	Tertile1 37.3 (≤53.3)	Tertile 2 66.0 (53.4–81.0)	Tertile3 103.0 (≥81.1)	
Model 1	Reference	1.022 (0.570–1.830)	2.957 (1.568–5.573)	<0.001
Model 2	Reference	1.136 (0.588–2.192)	3.133 (1.535–6.393)	0.001
Model 3	Reference	1.257 (0.634–2.489)	3.651 (1.722–7.741)	<0.001

Model 1: Unadjusted.

Model 2: Adjusted for age, sex, smoking, alcohol use, hypertension, DBP, history of stroke and complete occlusion of unilateral ICA.

Model 3: Adjusted for age, sex, smoking, alcohol use, hypertension, DBP, history of stroke and complete occlusion of unilateral ICA, MCA MFV and ROAF.

OR, odds ratio; CI, confidence interval; MCA, middle cerebral artery; PCA-P2, posterior cerebral artery P2 segment; MFV, mean flow velocity; ROAF, reversal of ophthalmic artery flow.

### 3.4 Association between ROAF and IS

The proportion of ROAF was higher in the stroke group (*p* = 0.004). After adjustment for confounding factors, ROAF was associated with IS (OR: 3.931, 95% CI: 1.764–8.760, *p* < 0.001).

### 3.5 Subgroup analysis: high-grade ICA stenosis versus complete ICA occlusion

To investigate whether severity of ICA lesion affects the robustness of the comparison of TCD hemodynamic parameters between the stroke group and the non-stroke group, this study conducted a sensitivity analysis. All included cases were divided into two subgroups—high-grade stenosis group and complete occlusion group, and a multivariate binary logistic regression analysis was performed again. The results showed that the correlation between MCA MFV and IS remained statistically significant in both subgroups (*p* = 0.049 and 0.011, respectively). PCA-P2 MFV was statistically significantly associated with IS only in the complete occlusion subgroup (*p* < 0.001). ROAF was statistically significantly associated with IS in the high-grade stenosis subgroup (*p* = 0.039), whereas the *p*-value was borderline in the complete occlusion group (*p* = 0.051).

## 4 Discussion

This study evaluated cerebral hemodynamics in patients with unilateral high-grade stenosis or occlusion of the ICA using TCD. We found that higher MCA BFV was associated with a reduced presence of IS in patients with unilateral high-grade stenosis or occlusion of the ICA; conversely, higher PCA-P2 BFV was associated with an increased presence of IS. ROAF was strongly associated with the presence of IS.

When the ICA flow is occluded, the circle of Willis allows a proportion of patients to remain asymptomatic. Therefore, the true prevalence of unilateral high-grade stenosis or occlusion of the ICA is unknown. A multicenter registry study in China included a total of 9,346 patients with IS, and 290 patients, or 3.1%, had ICA occlusion (9). Hemodynamic changes are the main cause of stroke in unilateral high-grade stenosis or occlusion of the ICA (10, 11). Even in the absence of IS, chronic hypoperfusion has a progressive impact on cognition (12).

Previous studies have shown that the more severe the ICA stenosis, the lower the ipsilateral MCA BFV; as the bilateral MCA BFV difference increased, the number of ischemic lesions increased (13). Mean MCA BFV correlates with late recovery of motor function after stroke in regions supplied by the MCA (14). MCA BFV is highly variable between patients. The lower the MCA BFV, the higher the incidence of stroke, so the need for prophylactic treatment should also be assessed promptly.

The anterior and posterior communicating arteries are the first collateral circulation to be initiated after an ICA blood flow deficit and are the main supply of the collateral circulation (2). The presence of leptomeningeal collateral activation in patients with chronic ICA occlusion indicates hemodynamic failure (15) and is a predictor of severely impaired cerebrovascular reserve function (16). On TCD, an increase in BFV of in the P2 segment indicates opening of the leptomeningeal collateral branches of the PCA. The increased BFV in PCA-P2 suggests the need to initiate as many collateral branches as possible to ensure blood supply. The question then arises: does the increase in PCA-P2 BFV actually relieve the ischemia? Or is it just an indicator of impaired hemodynamics?

In 2021, van Niftrik et al. (17) found that in symptomatic unilateral high-grade stenosis or occlusion of the ICA, increased PCA-P2 PSV was independently associated with blood oxygenation-level dependent-cerebrovascular reactivity-based hemodynamic impairment in the ipsilateral hemisphere, concluding that the potential clinical relevance of using TCD as a cost-effective screening tool for early hemodynamic impairment is very promising. Schneider et al. (16) concluded that increased PCA-P2 BFV on the affected side is an independent risk factor for stroke recurrence in patients with chronic ICA occlusion. Jud et al. (4) found that the risk of ipsilateral recurrent ischemic events in patients with symptomatic ICA occlusion could be predicted by a high PSV of PCA-P2. Our results are similar to the above findings, suggesting that even in unilateral high-grade stenosis or occlusion of the ICA without current ischemic symptoms, increased PCA-P2 BFV may be an indicator of urgent need for clinical intervention to prevent stroke.

ROAF is highly specific for TCD to detect ICA lesions (18, 19). Normal OA flow after ICA occlusion is an indication of the good condition of the main collateral branches (20). Several studies have concluded that OA collateralization is only indicative and has little effect on increasing intracranial blood supply (21, 22). Recently, it has also been suggested that ROAF may provide valuable but limited compensation when brain tissue is not adequately supplied with blood from the main lateral branches (23).

In unilateral high-grade stenosis or occlusion of the ICA, there is a pressure difference between the ICA and the ECA, and blood from the ECA will flow through the OA and its branches (including the STA) to the ICA, that is, the OA laterals will open. The incidence of stroke is higher in patients with ROAF, which is consistent with previous studies (18, 23). In recent years, the potential value of OA Doppler ultrasound in assessing acute stroke and cerebral hemodynamics has become clearer. As the largest branch of the ICA, the OA provides insight into intracranial circulation. In particular, the PSV/EDV ratio in the OA is recognized as an effective indicator of changes in cerebral perfusion. A recent prospective study by Kanter et al. (24) confirmed that OA Doppler ultrasound can distinguish between ischemic and hemorrhagic strokes at the bedside, with the PSV ratio achieving an AUC of 0.913, which highlighting the practical value of this technique in emergency settings where advanced neuroimaging is not readily available.

In a sensitivity analysis based on whether the internal ICA was completely occluded, the association between TCD parameters and IS differed between high-grade stenosis group and complete occlusion group (Table 5). MCA MFV was significantly associated with IS in both groups, suggesting that reduced BFV in the affected-side MCA is independently associated with IS. PCA-P2 MFV was significant only in the complete occlusion group, possibly because the drop in perfusion pressure is more pronounced during complete occlusion, requiring compensation from the posterior circulation (17). ROAF was significant in the high-grade stenosis group but only marginally significant in the complete occlusion group. In the context of an open anterior communicating artery, ROAF may reflect the additional recruitment of OA collateral pathways; its presence in the high-grade stenosis group may indicate that perfusion pressure has dropped to a level requiring the simultaneous activation of ECA-ICA collaterals, thereby exhibiting a stronger association with IS. In contrast, in the complete occlusion group, blood flow distal to the occlusion is severely compromised, and the strength of the association between IS and the additional activation of OA collaterals may consequently weaken.

**Table 5:** The multivariable binary logistic regression of TCD parameters for IS, by lesion severity (high-grade stenosis vs. complete occlusion).

Variables	High-grade stenosis		Complete occlusion	
	OR (95% CI)	*p*-value	OR (95% CI)	*p*-value
Age	1.035 (0.984–1.090)	0.180	1.041 (0.984–1.102)	0.158
Sex	0.772 (0.208–2.866)	0.699	1.659 (0.332–8.290)	0.537
Smoking	1.000 (0.408–2.453)	0.999	1.808 (0.557–5.863)	0.324
Alcohol use	4.576 (1.635–12.808)	0.004	2.565 (0.569–11.557)	0.220
Hypertension	2.166 (0.847–5.534)	0.106	0.837 (0.249–2.814)	0.773
SBP	1.005 (0.980–1.030)	0.693	0.986 (0.958–1.014)	0.315
DBP	1.004 (0.966–1.043)	0.848	1.098 (1.035–1.165)	0.002
History of stroke	-^*^	-^*^	3.375 (0.718–15.857)	0.123
T2DM	1.983 (0.844–4.663)	0.116	1.139 (0.376–3.455)	0.818
HDL-C	1.041 (0.893–1.213)	0.609	0.022 (0.002–0.245)	0.002
MCA MFV	0.977 (0.954–1.000)	0.049	0.948 (0.909–0.988)	0.011
PCA-P2 MFV	1.005 (0.993–1.017)	0.428	1.036 (1.016–1.057)	< 0.001
ROAF	3.516 (1.068–11.582)	0.039	3.327 (0.994–11.131)	0.051

OR, odds ratio; CI, confidence interval; SBP, systolic blood pressure; DBP, diastolic blood pressure; T2DM, type 2 diabetes mellitus; HDL-C, high density lipoprotein cholesterol; MCA, middle cerebral artery; PCA-P2, posterior cerebral artery P2 segment; MFV, mean flow velocity; ROAF, reversal of ophthalmic artery. * Complete separation occurred; OR and 95% CI were unstable and thus not included in the final model.

TCD has the disadvantage of not being able to show vessel morphology and its accuracy depends on the experience of the operator. This is also a limitation of this study. Additionally, the majority of TCD examinations in this retrospective study were performed by a single operator, and intraobserver and interobserver reliability were not assessed. We acknowledge this as a limitation when interpreting our findings.

The optimal timing and strategy for surgical intervention in severe unilateral ICA stenosis remains to be determined (25). Our results suggest that cerebral hemodynamics are closely related to the presence of IS in patients with unilateral high-grade stenosis or occlusion of the ICA. TCD parameters may help identify unfavorable hemodynamic patterns, but prospective studies are needed before using them to guide treatment timing.

## 5 Conclusion

Although patients with the same lesion – high-grade stenosis or occlusion of the ICA – experience different outcomes in terms of whether they have an IS, these outcomes are closely related to their cerebral hemodynamic status. Hemodynamic parameters measured by TCD are closely associated with stroke occurrence in these patients.

## Data Availability

The raw data supporting the conclusions of this article will be made available by the authors, without undue reservation.
